# Differentiating neurosarcoidosis from multiple sclerosis using combined analysis of basic CSF parameters and MRZ reaction

**DOI:** 10.3389/fneur.2023.1135392

**Published:** 2023-03-24

**Authors:** Benjamin Vlad, Stephan Neidhart, Marc Hilty, Mario Ziegler, Ilijas Jelcic

**Affiliations:** ^1^Department of Neurology, University Hospital Zurich and University of Zurich, Zurich, Switzerland; ^2^Neuroimmunology and Multiple Sclerosis Research Section, Department of Neurology, University Hospital Zurich and University of Zurich, Zurich, Switzerland

**Keywords:** neurosarcoidosis, multiple sclerosis, cerebrospinal fluid, pleocytosis, blood-CSF barrier dysfunction, oligoclonal bands, MRZ reaction

## Abstract

**Background:**

Neurosarcodosis is one of the most frequent differential diagnoses of multiple sclerosis (MS) and requires central nervous system (CNS) biopsy to establish definite diagnosis according to the latest consensus diagnostic criteria. We here analyzed diagnostic values of basic cerebrospinal fluid (CSF) parameters to distinguish neurosarcoidosis from MS without CNS biopsy.

**Methods:**

We retrospectively assessed clinical, radiological and laboratory data of 27 patients with neurosarcoidosis treated at our center and compared following CSF parameters with those of 138 patients with relapsing-remitting MS: CSF white cell count (WCC), CSF/serum albumin quotient (Q_alb_), intrathecal production of immunoglobulins including oligoclonal bands (OCB), MRZ reaction, defined as a polyspecific intrathecal production of IgG reactive against ≥2 of 3 the viruses measles (M), rubella (R), and zoster (Z) virus, and CSF lactate levels. Additional inflammatory biomarkers in serum and/or CSF such as neopterin, soluble interleukin-2 receptor (sIL-2R) and C-reactive protein (CRP) were assessed.

**Results:**

There was no significant difference in the frequency of CSF pleocytosis, but a CSF WCC > 30/μl was more frequent in patients with neurosarcoidosis. Compared to MS, patients with neurosarcoidosis showed more frequently an increased Q_alb_ and CSF lactate levels as well as increased serum and CSF levels of sIL-2R, but a lower frequency of intrathecal IgG synthesis and positive MRZ reaction. Positive likelihood ratio (PLR) of single CSF parameters indicating neurosarcoidosis was highest, if (a) CSF WCC was >30/μl (PLR 7.2), (b) Q_alb_ was >10 × 10^−3^ (PLR 66.4), (c) CSF-specific OCB were absent (PLR 11.5), (d) CSF lactate was elevated (PLR 23.0) or (e) sIL-2R was elevated (PLR>8.0). The combination of (a) one of three following basic CSF parameters, i.e., (a.1.) CSF WCC >30/ul, or (a.2.) Q_Alb_ >10 × 10^−3^, or (a.3.) absence of CSF-specific OCB, and (b) absence of positive MRZ reaction showed the best diagnostic accuracy (sensitivity and specificity each >92%; PLR 12.8 and NLR 0.08).

**Conclusion:**

Combined evaluation of basic CSF parameters and MRZ reaction is powerful in differentiating neurosarcoidosis from MS, with moderate to severe pleocytosis and Q_Alb_ elevation and absence of intrathecal IgG synthesis as useful rule-in parameters and positive MRZ reaction as a rule-out parameter for neurosarcoidosis.

## 1. Introduction

Sarcoidosis is an inflammatory granulomatous disease, affecting the central nervous system (CNS) in 5–15% of cases, then referred to as neurosarcoidosis. The prevalence of neurosarcoidosis (~3–4/100'000) is much lower than multiple sclerosis (MS), which is by far the most frequent inflammatory CNS disease (190/100'000 in Switzerland, global burden 44/100'000 with prevalence ranging from 4.8/100'000 in Western Pacific countries to 117/100'000 in Americas and 143/100'000 in European countries) and the most frequently evaluated differential diagnosis (1–5). However, neurosarcoidosis is most probably more frequent than other neuroimmunological diseases mimicking MS such as neuromyelitis optica spectrum disorder (NMOSD, prevalence ~1/100'000) or MOG antibody-associated disease (MOGAD, prevalence ~2/100'000) (6–10). While evidence of autoimmune pathomechanisms in sarcoidosis is accumulating, there is yet no consensus about its definition as an autoimmune disease and no clear understanding, if immunologic pathways of systemic sarcoidosis, e.g., pulmonary sarcoidosis, can be translated to neurosarcoidosis (11). A mixture of complex genetic patterns and different environmental exposures appear to contribute to the risk of disease development (12–16). Neurologic involvement is, among several other factors, associated with increased mortality in sarcoidosis, but establishing the diagnosis of neurosarcoidosis remains challenging (11, 17). Due to its diverse clinical phenotypes, differentiating neurosarcoidosis from other inflammatory CNS diseases, e.g., MS, can cause difficulties, but is important for the selection of effective treatment strategies. Furthermore, latest consensus diagnostic criteria stipulate that only CNS biopsy with histopathological evidence can establish the diagnosis of definite neurosarcoidosis (18, 19). However, it would be favorable in the future to develop a reliable combination of cerebrospinal fluid (CSF) parameters, radiological and clinical features that would obviate the need for an invasive biopsy.

Recent studies indicate absence of CSF-specific oligoclonal bands (OCB) as a good CSF marker to distinguish neurosarcoidosis from MS, which is the most frequently evaluated differential diagnosis (20). However, basic CSF diagnostics involving CSF white cell count and profiling, blood CSF barrier function, CSF lactate and serum/CSF glucose ratio appear to help distinguishing both disease entities, and data about positive and negative likelihood ratios (PLR and NLR) of single parameters or the combination thereof to help in diagnosing neurosarcoidosis is scarce. We here extended the analysis of basic CSF parameters in both diseases to the MRZ reaction, which is defined as a polyspecific intrathecal production of IgG reactive against ≥2 of 3 the viruses measles (M), rubella (R), and zoster (Z) virus and which is reported as a highly specific CSF biomarker of MS.

## 2. Material and methods

### 2.1. Patients

We retrospectively analyzed clinical, radiological and laboratory data from 27 patients with possible (*n* = 4), probable (*n* = 19), or definite (*n* = 4) neurosarcoidosis according to consensus diagnostic criteria (19) for predominant clinical phenotype, MRI, FDG-PET and CT findings, CNS or lymph node biopsy locations (Supplementary Table 1) and, if available, frequency of pathologic inflammatory serum and/or CSF markers (Tables 1, 2). In a second step, basic demographic and CSF data from neurosarcoidosis patients and 138 patients with untreated relapsing-remitting multiple sclerosis (MS) were compared for CSF white cell count (WCC), CSF/serum albumin ratio (Q_Alb_), IgG, IgA and IgM CSF/serum ratios (Q_IgG_, Q_IgA_, Q_IgM_), frequency of intrathecal synthesis of IgG, IgA, and IgM according to Reiber's diagram (21) and their intrathecal fraction (IgG_IF_, IgA_IF_, and IgM_IF_), frequency of CSF-specific OCB, intrathecal production of IgG reactive against measles, rubella and varicella zoster virus (MRZ reaction), CSF lactate levels and CSF/serum glucose ratio. 25/27 (92.6%) neurosarcoidosis patients were untreated at the time of lumbar puncture, 1 patient with possible neursarcoidosis was under treatment with mycophenolate mofetil, 1 patient with definite neurosarcoidosis was under treatment with methotrexate. Informed consent was obtained from all patients. Since data of all patients were anonymized for this study, the local Cantonal Ethics Committee stated that the research project does not fall within the scope of the Human Reseach Act (HRA) and therefore, an authorization from the ethics committee is not required (BASEC Nr. Req-2022-01134).

**Table 1:** Clinical and radiological characteristics of neurosarcoidosis patients.

**Characteristics**	***n*/*N* (%)**
**Diagnostic classification by 2018 consensus diagnostic criteria (**19**)**	
- Possible neurosarcoidosis	4/27 (14.8%)
- Probable neurosarcoidosis	19/27 (70.4%)
- Definite neurosarcoidosis	4/27 (14.8%)
**Neurological manifestation before detection of systemic sarcoidosis**	19/27 (70.4%)
- among cases with possible neurosarcoidosis	4/19 (21.1%)
- among cases with probable neurosarcoidosis	11/19 (57.9%)
- among cases with definite neurosarcoidosis	4/19 (21.1%)
**Extraneuronal manifestation (systemic involvement)**	
- Lymphadenopathy^*^	24/27 (88.9%)
- Lung	21/27 (77.8%)
- Bone	8/27 (29.6%)
- Skin	4/27 (14.8%)
- Kidney	4/27 (14.8%)
- Heart	3/27 (11.1%)
- Liver	2/27 (7.4%)
- Glands	2/27 (7.4%)
- Spleen	2/27 (7.4%)
- Eye	2/27 (7.4%)
**First clinical event**	
- Sensory and/or motoric dysfunction	6/27 (22.2%)
- Myelitis	6/27 (22.2%)
- Optic neuritis	4/27 (14.8%)
- Gait disturbance	3/27 (11.1%)
- Meningoencephalitis	3/27 (11.1%)
- Extraocular movement impairment	2/27 (9.1%)
- Vestibular syndrome	2/27 (9.1%)
- Cerebellar syndrome	1/27 (3.7%)
**Radiographic phenotype**	
- Supratentorial manifestation	19/27 (70.4%)
- Leptomeningeal enhancement	16/27 (59.3%)
- Infratentorial manifestation	12/27 (44.4%)
- Spinal manifestation	6/27 (22.2%)
- No abnormality	3/27 (11.1%)
**FDG-PET scan**	
- Sarcoidosis-like systemic lesions and/or abnormality^**^	15/16 (93.8%)

* Lymphadenopathy was counted, if lymph nodes at characteristic anatomic sites were (i) metabolically active in FDG-PET and/or (ii) enlarged in FDG-PET or CT scan of the thorax, abdomen or neck.

** Sarcoidosis-like systemic lesions involved mainly metabolically active (i) pulmonary hilar lymph nodes and/or (ii) extrapulmonary, i.e., cervical, mesenterial and inguinal, lymph nodes as identified by FDG-PET scan, but also less frequntly metabolically active lesions within extraneuronal organs as listed under above mentioned “extraneuronal manifestation (systemic involvement)”.

**Table 2:** Prevalence of pathological inflammatory serum and/or CSF markers in neurosarcoidosis patients.

**CSF or serum marker**	**Normal value**	**Prevalence of patients with elevated marker, *n*/*N* (%)**
Serum neopterin elevated	<2.5 ng/ml	17/20 (85.0%)
CSF neopterin elevated	<1.5 ng/ml	5/5 (100.0%)
CSF neopterin and/or serum neopterin elevated	See above	19/22 (86.4%)
CSF neopterin elevated, serum neopterin not elevated	See above	2/5 (40.0%)
Serum sIL-2R elevated	<477 pg/ml	10/25 (40.0%)
CSF sIL-2R CSF elevated	<150 pg/ml	9/21 (42.9%)
CSF sIL-2R and/or serum sIL-2R elevated	See above	16/25 (64.0%)
CSF sIL-2R elevated, serum sIL-2R not elevated	See above	6/21 (28.6%)
Serum TNF-α elevated	<12.0 pg/ml	10/19 (52.6%)
Serum β_2_ microglobulin elevated	<2.5 mg/l	4/8 (50.0%)
Serum IL-6 elevated	<3.0 pg/ml	8/18 (44.4%)
Serum ACE elevated	19.8–70.2 U/l	6/20 (30.0%)
Serum CRP elevated	<5 mg/l	8/27 (29.6%)
Serum IL-1β elevated	<2.0 pg/ml	0/14 (0.0%)

### 2.2. Inflammatory serum and CSF markers

Neurosarcoidosis patients were screened for data availability of serum neopterin (normal value <2.5 ng/ml, neopterin ELISA kit, IBL International), CSF neopterin [normal value <1.5 ng/ml according to Motta et al. (22)], serum soluble interleukin-2 receptor (sIL2-R, normal value <477 pg/ml, sIL-2R ELISA kit, R&D Biotech), CSF sIL2R [normal value <150 pg/ml according to Petereit et al. (23)], serum tumor necrosis factor alpha (TNF-α, normal value <12.0 pg/ml, TNF-alpha ELISA kit, IBL International), serum β_2_ microglobulin (normal value <2.5 mg/l, Beta-2 microglobulin Optilite Kit, Binding Site), serum interleukin 6 (IL-6, normal value <3.0 pg/ml, Interleukin-6 ELISA kit, IBL International), serum interleukin-1 beta (IL-1β, normal value <2.0 pg/ml, Interleukin-1beta ELISA kit, IBL international), serum angiotensin-converting enzyme [ACE, normal value 19.8–70.2 U/l according to Lopez-Sublet et al. (24)] and C-reactive peptide [CRP, normal value <5 mg/l according to Eda et al. (25)] and the frequencies of respective pathologic values were captured. Additionally, patients with neurosarcoidosis and MS were compared for prevalence of elevated serum sIL-2R, CSF sIL-2R and serum CRP.

### 2.3. Cytological examination and clinical chemistry of CSF

A CSF white cell count >4/μl was classified as “increased,” representing pleocytosis. WCC was further grouped into (i) 0-−4/μl (no pleocytosis), (ii) 5–30/μl (mild pleocytosis) and (iii) >30/μl (moderate pleocytosis) as an assessment of pleocytosis severity. CSF lactate was considered pathologic if outside of range 1.7–2.6 mmol/l. CSF/blood ratio of glucose was calculated, a ratio <0.5 was considered pathologic.

### 2.4. Evaluation of blood-CSF barrier function and intrathecal production of immunoglobulins

Albumin levels and IgG, IgM and IgG levels in CSF and serum were quantified by immunonephelometry (Atellica NEPH 630 System, Siemens Healthineers, Switzerland) and respective CSF/serum ratios were calculated. Function of the blood-CSF barrier (BCSFB) was assessed using CSF/serum albumin quotient (Q_Alb_), with Q_Alb_ = CSF albumin concentration / serum albumin concentration. The upper reference limit of Q_Alb_ was calculated as Q_Alb_ = [4+(age/15)] × 10^−3^ according to Reiber et al. (26), with age representing the patient's age in years. Elevated Q_Alb_ indicated disturbed BCSFB, with Q_Alb_ >10 × 10^−3^ defined as moderate BCSFB dysfunction and Q_Alb_ >20 × 10^−3^ defined as severe BCSFB disruption.

IgG index was calculated as Q_IgG_/Q_Alb_, with Q_IgG_ = CSF IgG concentration / serum IgG concentration. IgG index ≥0.7 indicated intrathecal synthesis of IgG. The relative intrathecal fraction of IgG, IgA, and IgM (IgG_IF_, IgA_IF_, and IgM_IF_) were, respectively calculated according to Reiber (21). IgG_IF_ >0%, IgA_IF_ >0% and/or IgM_IF_ >0% indicated intrathecal synthesis of IgG, IgA, and/or IgM, respectively. OCB were detected by isoelectric focusing (IEF) on agarose gels and immunoblotting using IgG-specific antibodies in a semi-automated approach (Interlab G26, Interlab, Italy). OCB patterns were evaluated according to international consensus criteria (27): OCB pattern type 1 = no OCBs in CSF or Serum; OCB pattern type 2 = CSF-restricted OCBs (intrathecal IgG production); OCB pattern type 3 = identical bands in CSF and serum (i.e., systemic inflammation), and additional CSF-restricted OCBs (i.e., intrathecal IgG production); OCB pattern type 4 = identical OCBs in CSF and serum; and OCB pattern type 5 = monoclonal bands in CSF and serum. Intrathecal IgG synthesis was indicated only by IEF patterns 2 and 3. OCBs were considered CSF-restricted, if ≥2 additional bands were detected in CSF compared to serum.

### 2.5. MRZ reaction

Measles- (M), rubella- (R) and varicella zoster (Z) virus-specific IgG antibodies were measured in paired CSF and serum samples with commercial ELISA kits and fully automated ELISA processing. Serum (1:404 and 1:2,020 or 1:3,232 dilutions) and CSF (1:2 and 1:10, or 1:8, 1:16, 1:40, 1:80 dilutions) were analyzed using commercial ELISA kits (EI 2610-9601-L G; EI 2590-9601-LG; EI 2650-9601-L G) from Euroimmun AG (Switzerland) and fully automated ELISA processing (Analyzer I, Euroimmun AG, Switzerland).

The virus-specific CSF/serum antibody index (CAI_spec_) was calculated as previously described by Reiber (21). In short, CAI_spec_ was assessed as CAI_spec_ = Q_spec_/Q_IgG_ (if Q_Lim_ (IgG) > Q_IgG_), or CAI_spec_ = Q_spec_/Q_Lim_ (IgG), if Q_Lim_ (IgG) < Q_IgG_. The respective parameters were calculated as follows: Q_spec_ = antigen-specific IgG_CSF_ [AU]/antigen-specific IgG_serum_ [AU]; Q_IgG_ = total IgG_CSF_ [mg/l]/total IgG_serum_ [mg/l]; Q_Lim_ (IgG) = 0.93 × (${\text{Q}}_{\text{Alb}}^{2}$ + 6 × 10^−6^)^0.5^ – 1.7 × 10^−3^; Q_Lim_ (IgG) refers to the upper discrimination line of the hyperbolic reference range for the blood-derived IgG in CSF as absence of intrathecal IgG synthesis. CAI_spec_ ≥1.5 indicated intrathecal synthesis of virus-specific antibodies. MRZ reaction (MRZR) was interpreted as positive, if polyclonal intrathecal production of antibodies against ≥2 of the 3 antigens measles (M)-, rubella (R)- and varicella zoster (Z) virus lysate, was detectable (28).

### 2.6. Statistics

Differences in age and disease duration were compared with the Kruskal-Wallis test. Differences in frequency of female gender, pleocytosis, elevated Q_Alb_, intrathecal immunoglobulin synthesis according to Reiber, CSF-specific OCB, elevated CSF lactate, pathologic CSF/serum glucose ratio, elevetad sIL-2R levels in serum or CSF, elevated CRP levels in serum and positive MRZ reaction were compared with Yates's chi-squared test. Differences in mean values of WCC, Q_Alb_, immunoglobulin CSF/serum ratios, IgG index and intrathecal fraction of immunoglobulins were calculated using unpaired *t*-test. Sensitivity, specificity, positive likelihood ratio (PLR) and negative likelihood ratio (NLR) with 95%-confidence intervals (95%-CI) of single or combined basic CSF/serum parameters were analyzed to assess their value in distinguishing neurosarcoidosis from MS.

## 3. Results

First clinical symptoms of neurosarcoidosis patients included optic neuritis (14.8%), sensory and/or motoric dysfunction (22.2%), myelitis (22.2%), extraocular movement impairment (9.1%), gait disturbance (11.1%), cerebellar syndrome (3.7%), vestibular syndrome (9.1%), and meningoencephalitis (11.1%), strongly mimicking MS-like CNS syndromes in 81.5% of cases (Table 1). 19/27 (70.4%) neurosarcoidosis patients had neurological manifestation before detection of systemic sarcoidosis (Table 1). 88.9% of neurosarcoidosis patients showed MRI abnormalities, mostly involving leptomeningeal enhancement (Table 1). Of those patients who received an FDG-PET scan, 93.4% showed sarcoidosis-typical systemic lesions and/or abnormalities (Table 1).

Analysis of inflammatory markers in serum and/or CSF of neurosarcoidosis patients showed elevated levels of neopterin in CSF or serum in 19/22 (86.4%) patients, elevated levels of sIL-2R in CSF or serum (16/25, 64.0%), elevated serum TNF-α (10/19, 52.6%), elevated serum β_2_ microglobulin (4/8, 50.0%), elevated serum IL-6 (8/18, 44.4%%), elevated serum ACE (6/20, 30.0%) and elevated serum CRP (8/27, 29.6%) in descending order, while no patient showed elevated levels of IL-1β (0/14, 0.0%) (Table 2). Elevated neopterin levels in serum were detected in 17/20 (85.0%) patients, and elevated neopterin levels in CSF in 5/5 (100.0%) patients, of whom 2/5 (40.0%) had only elevated CSF neopterin levels with normal serum neopterin levels. Elevated levels of sIL-2R in serum were detected in 10/25 (40.0%) patients, and elevated levels of sIL-2R in CSF in 9/21 (42.9%), of whom 6/21 (28.6%) had only elevated CSF sIL-2R, but normal sIL-2R levels in serum (Table 2).

Comparison of demographic features (Table 3) showed that patients with neurosarcoidosis (median age 43.5 years, interquartile range [IQR] 40–51.5) were significantly older at time of lumbar puncture than MS patients (median age 32 years, IQR 28-38; *p* < 0.001) and had a longer disease duration in months from first clinical presentation until lumbar puncture (6.0 months, IQR 0.0–15.8 vs. 0.0, IQR 0.0–2.0; *p* = 0.013). Furthermore, female sex was more frequent in MS than in neurosarcoidosis (67.4 vs. 44.4%; *p* = 0.041, Table 3).

**Table 3:** Demographic features of patients with neurosarcoidosis and MS.

**Parameter**	**Overall**	**Neurosarcoidosis**	**MS**	***p*-value**
*n*	165	27	138	
Female gender, n (%)	105 (63.6%)	12 (44.4%)	93 (67.4%)	**0.041**
Age at LP, median [IQR]	34.0 [28.0–42.0]	43.5 [40.0–51.5]	32.0 [28.0–38.0]	**<0.001**
Disease duration between first symptoms and lumbar puncture in months, median [IQR]	0.0 [0.0–5.2]	6.0 [0.0–15.8]	0.0 [0.0–2.0]	**0.013**
Disease duration between first symptoms and lumbar puncture in years, mean (SD)	1.4 (3.9)	2.6 (5.2)	1.1 (3.4)	0.218

LP, lumbar puncture. The bold values indicate statistically significant results (*p* < 0.05).

There was no significant difference in the frequency of pleocytosis (63.8 vs. 55.6%; *p* = 0.42) between patients with neurosarcoidosis and patients with MS, but a CSF WCC >30/μl was more frequent in patients with neurosarcoidosis (22.2 vs. 3.6%; *p* < 0.001) and a CSF WCC >100/μl was present in neurosarcoidosis patients only (7.4 vs. 0.0%; *p* = 0.001) (Table 4). These findings corroborate previous reports from the current literature, where 31.8–62.7% of patients with definite or probable neurosarcodosis had CSF pleocytosis among studies involving more than 20 patients (Supplementary Table 2) (18, 20, 29, 30).

**Table 4:** Comparison of basic CSF parameters between patients with neurosarcoidosis or MS.

**CSF parameter**	**Overall**	**Neurosarcoidosis**	**MS**	***p*-value**
**Pleocytosis**^**a**^, ***n*** **(%)**	103 (62.4%)	15 (55.6%)	88 (63.8%)	0.5562
CSF WCC (cells/μl), mean (SD)	-	39.2 (108.8%)	9.2 (10.5%)	0.017
- WCC 5–30/μl, *n* (%)	92 (55.8%)	8 (29.6%)	83 (60.1%)	**0.0068**
- WCC >30/μl, *n* (%)	11 (6.7%)	6 (22.2%)	5 (3.6%)	**0.0018**
- WCC >100/μl, *n* (%)	2 (1.2%)	2 (7.4%)	0 (0.0%)	**0.0241**
**BCSFB disruption**^**b**^, ***n*** **(%)**	55 (33.3%)	19 (70.4%)	36 (26.1%)	**<0.001**
Q_Alb_ (×10^−3^), mean (SD)	6.7 (7.8%)	16.5 (16.7%)	5.0 (1.8%)	**<0.001**
- Q_Alb_ >10 × 10^−3^	14 (8.5%)	13 (48.1%)	1 (0.7%)	**<0.001**
- Q_Alb_ >20 × 10^−3^	7 (4.2%)	7 (25.9%)	0 (0.0%)	**<0.001**
**Intrathecal immunoglobulin synthesis** ^**c**^				
IgG index, mean (SD)	0.9 (0.5%)	0.6 (0.3%)	0.9 (0.5%)	**0.0022**
IgG_IF_ >0%, n (%)	74 (45.1%)	3 (7.7%)	72 (52.2%)	**<0.001**
IgA_IF_ >0%, *n* (%)	16 (9.8%)	5 (15.4%)	12 (8.7%)	0.234
IgM_IF_ >0%, *n* (%)	27 (16.5%)	5 (15.4%)	23 (16.7%)	0.815
CSF-specific OCB, *n* (%)	139 (84.2%)	9 (33.3%)	130 (94.2%)	**<0.001**
**Elevated CSF lactate**^**d**^, ***n*** **(%)**	11 (6.7%)	9 (33.3%)	2 (1.4%)	**<0.001**
**Pathologic CSF/serum glucose ratio**^**e**^, ***n*** **(%)**	7 (4.3%)	7 (26.9%)	0 (0.0%)	**<0.001**

a Normal value WCC: 0–4 cells/μl.

b Normal value BCSFB, i.e., Q_Alb_: < [4+(age/15)] × 10^−3^.

c Normal value IgG index: < 0.7; normal value IgG_IF_ < 0%, normal value IgA_IF_ < 0%, normal value IgM_IF_ < 0%, i.e. no intrathecal immunoglobulin synthesis.

d Normal value CSF lactate: 1.7–2.6 mmol/l.

e Normal value CSF/serum glucose ratio: ≥ 0.5.

BCSFB, blood CSF barrier; OCB, oligoclonal IgG bands; WCC, white cell count. The bold values indicate statistically significant results (*p* < 0.05).

Elevated Q_Alb_ indicating BCSFB dysfunction showed a much stronger association with neurosarcoidosis than with MS (70.4 vs. 26.1%; *p* < 0.001) (Table 4). This association was even stronger with Q_Alb_ >10 × 10^−3^ (48.1 vs. 0.7%, *p* < 0.001). A severe elevation of Q_Alb_ >20 × 10^−3^ was again present in neurosarcoidosis patients only (25.9 vs. 0.0%, *p* < 0.001). This finding matches well with the current literature, which reports increased CSF protein levels as alternative measure of BCSFB dysfunction in 45.5–76.4% (Supplementary Table 2) (18, 20, 29, 30).

Intrathecal synthesis of IgG according to Reiber's diagram was more frequent in MS patients (52.2 vs. 7.7%, *p* < 0.001), whereas no significant difference in intrathecal synthesis of IgA or IgM, respectively, was detected (Table 4). Neurosarcoidosis patients showed higher values in CSF/serum IgG ratio (9.3 [SD 10.2] vs. 4.5 [SD 2.5], *p* = 0.024) and IgA ratio (8.9 [13.5%] vs. 1.8 [1.9], *p* = 0.014), but not IgM ratio (4.1 [9.9] vs. 0.7 [1.1], *p* = 0.093), than MS patients (Supplementary Table 3). However, these findings should be interpreted in line with the more frequent and more intense BCSFB dysfunction observed in neurosarcoidosis patients, since CSF/serum immunoglobulin ratios are biased by Q_Alb_. Interestingly, if intrathecal synthesis of IgG, IgA or IgM was present, the intensity of production, described as relative intrathecal fraction Ig_IF_ according to Reiber (21), did not vary between neurosarcoidosis and MS (Supplementary Table 3). CSF-specific OCB were present in most of the MS patients (94.2%), but also in 33.3% of the neurosarcoidosis patients (*p* < 0.001) (Table 4). The latter finding is higher than in recent studies [2.9–4.5% in Arun et al. (20) and Kidd (30)], but in line with older studies [27.3–37.0% in Joseph and Scolding (29) and in Zajicek et al. (18)] (Supplementary Table 2).

Furthermore, patients with neurosarcoidosis were significant more likely to show pathological levels of CSF lactate (33.3 vs. 1.4%, *p* < 0.001) and pathological CSF/serum glucose ratio (26.9 vs. 0%, *p* < 0.001) (Table 4).

A positive MRZ reaction was present in 33.3% of MS patients, but only in a single patient with neurosarcoidosis (3.7%, *p* < 0.001) (Table 5). Significant differences in the prevalence of IgG synthesis reactive against measles virus antigens (3.7 vs. 30.4%, *p* = 0.004), rubella virus antigens (11.1 vs. 39.1%, *p* = 0.005) and varicella zoster virus antigens (7.4 vs. 50.7%, *p* < 0.001) were detected between neurosarcoidosis patients and MS patients (Table 5). Not only the single virus-specific antibody reactivity species (measles or rubella or zoster), but also most of the combinations thereof were significantly less frequent in neurosarcoidosis than in MS patients (Supplementary Table 4). The rubella- and zoster-specific CAI values, but not measles-specific CAI values, were significantly lower in neurosarcoidosis patients compared to MS patients (Supplementary Table 5). These results are in line with two previous studies, which reported a positive MRZ reaction in < 1–9.1% of patients (Supplementary Table 6) (31, 32). However, after including single cases reporting MRZ reaction in patients neurosarcoidosis, the overall frequency of positive MRZ reaction was 3/27 (11.1%) (Supplementary Table 6) (28, 33–35).

**Table 5:** Comparison of frequency of MRZ reaction in patients with neurosarcoidosis and patients with MS.

**Parameter**	**Overall**	**Neurosarcoidosis**	**MS**	***p*-value**
Intrathecal measles-specific IgG production (M), *n*/*N* (%)	43/165 (26.1%)	1/27 (3.7%)	42/138 (30.4%)	0.0088
Intrathecal rubella-specific IgG production (R), *n*/*N* (%)	56/165 (33.9%)	2/27 (7.4%)	54/138 (39.1%)	0.0031
Intrathecal zoster-specific IgG production (Z), *n*/*N* (%)	71/165 (43.0%)	1/27 (3.7%)	70/138 (50.7%)	<0.001
Positve MRZ reaction^a^, *n*/*N* (%)	47/165 (28.5%)	1/27 (3.7%)	46/138 (33.3%)	0.0039

a positive MRZ reaction defined as intrathecal production of IgGs reactive against at least two of three antigens (M, R and Z), i.e., M+R or M+Z or R+Z or M+R+Z.

**Figure 1:**
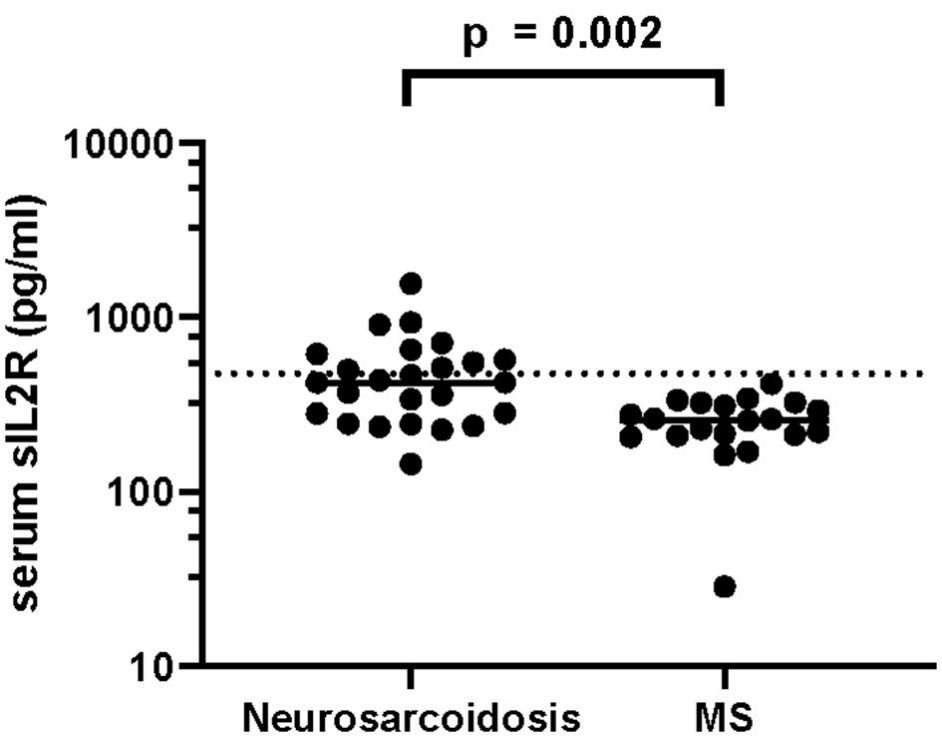
Serum sIL-2R levels in neurosarcoidosis and MS patients. The dashed line represents the cut-off for normal values.

**Figure 2:**
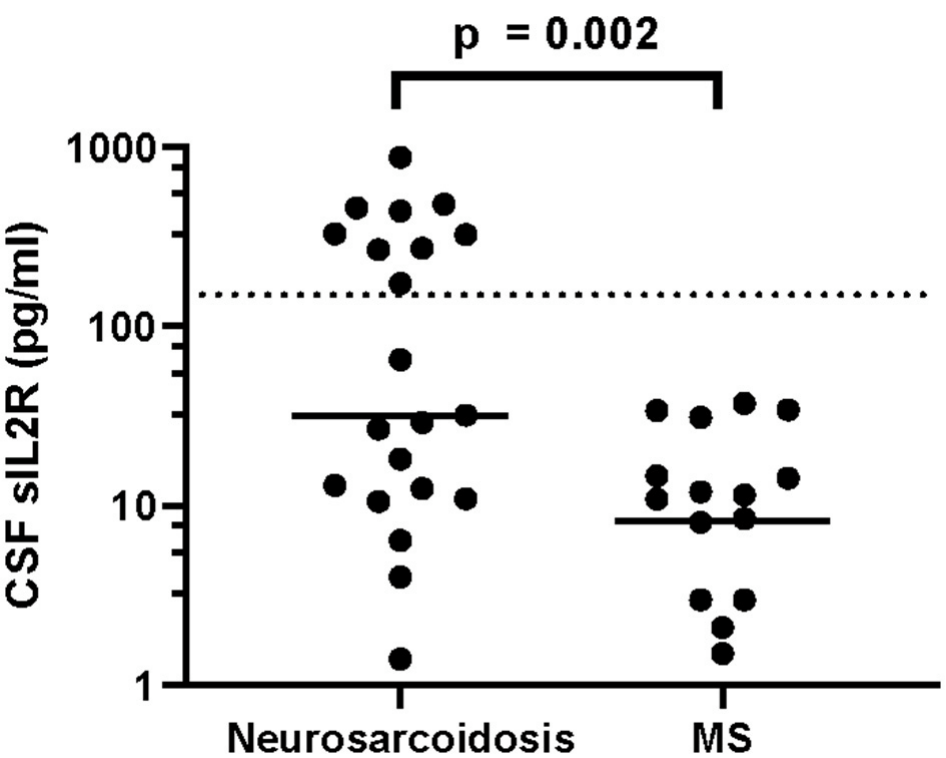
CSF sIL-2R levels in neurosarcoidosis and MS patients. The dashed line represents the cut-off for normal values.

**Figure 3:**
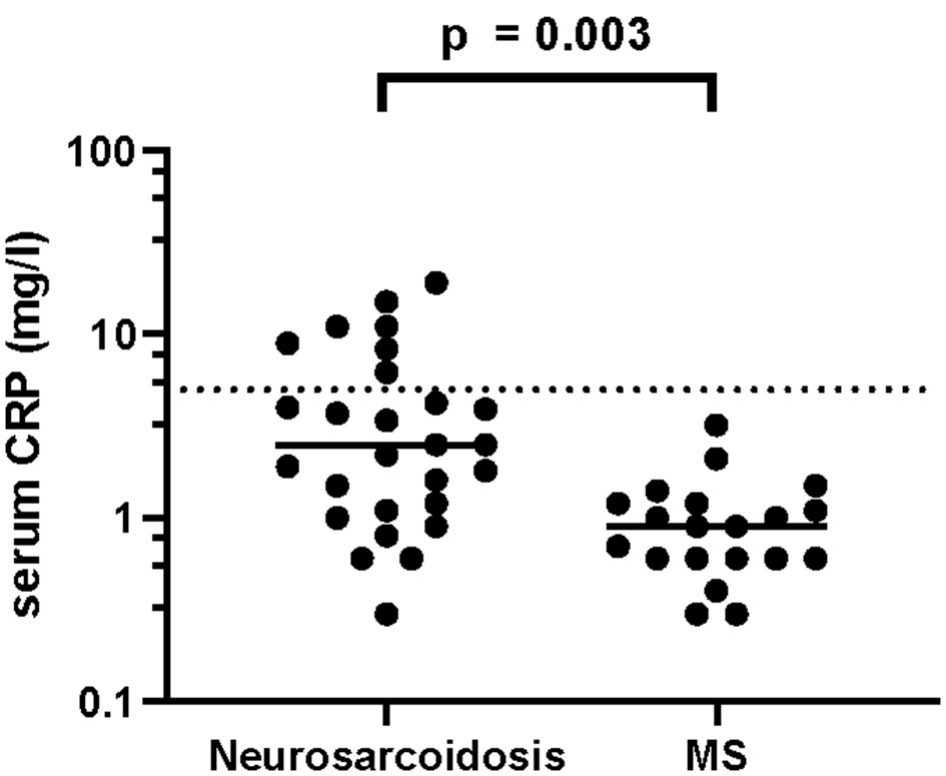
CSF CRP levels in neurosarcoidosis and MS patients. The dashed line represents the cut-off for normal values.

Elevated levels of sIL-2R in serum (40.0 vs. 5.0%, *p* = 0.007) and CSF (38.1 vs. 0.0%, *p* = 0.002) as well as elevated serum CRP (25.9 vs. 0.0%, 0.014) showed a strong association with neurosarcoidosis compared to MS (Table 6). Accordingly, serum sIL-2R levels (Figure 1), CSF sIL-2R levels (Figure 2) and serum CRP levels (Figure 3) were significantly higher in neurosarcoidosis than in MS patients.

**Table 6:** Comparison of serum sIL-2R, CSF sIL-2R and serum CRP levels between patients with neurosarcoidosis and patients with MS.

**CSF/serum markers**	**Overall**	**Neurosarcoidosis**	**MS**	***p*-value**
Frequency of elevated serum sIL-2R levels, n/N (%)	11/45 (24.4%)	10/25 (40.0%)	1/20 (5.0%)	0.018
- Serum sIL-2R levels, mean (SD)	384.4 (256.5)	489.4 (303.3)	253.0 (83.2)	0.0016
Frequency of elevated CSF sIL-2R levels, n/N (%)	8/41 (19.5%)	8/21 (38.1%)	0/20 (0.0%)	0.0073
- CSF sIL-2R levels, mean (SD)	99.4 (184.8)	183.4 (233.9)	11.3 (12.8)	0.0022
Frequency of elevated serum CRP levels, *n*/*N* (%)	7/47 (14.9%)	7/27 (25.9%)	0/20 (0.0%)	0.04
- Serum CRP levels, mean (SD)	3.0 (4.0)	4.4 (4.8)	1.0 (0.7)	0.003

In order to distinguish neurosarcoidosis from MS by laboratory parameters, we further analyzed sensitivities and specificities, as well as positive and negative likelihood ratios (PLR and NLR) of single inflammatory markers and basic CSF parameters (Table 7) and the combinations thereof (Table 8). Diagnostic tests with a PLR of >10 and NLR < 0.1 are generally considered as useful (36, 37), since they are increasing the probability for a defined condition by ~45% (38).

**Table 7:** Sensitivity, specificity, and likelihood ratios of single inflammatory markers and basic CSF parameters to distinguish neurosarcoidosis from MS.

**Parameter**	**Neuro-sarcoidosis, *n*/*N* (%)**	**MS, *n*/*N* (%)**	**Sensitivity, % (95% CI)**	**Specificity, % (95% CI)**	**PLR (95% CI)**	**NLR (95% CI)**
1. Serum sIL-2R elevated	10/25 (40.0%)	1/20 (5.0%)	40.0% (20.8–59.2%)	95.0% (85.4–100.0%)	8.00 (1.12–57.35)	0.632 (0.45–0.88)
2. CSF sIL-2R elevated	8/21 (38.1%)	0/20 (0.0%)	38.1% (17.3–58.9%)	100.0%	-	0.619 (0.44–0.87)
3. Serum CRP elevated	7/27 (25.9%)	0/20 (0.0%)	25.9% (9.4–42.4%)	100.0%	-	0.741 (0.83–1.00)
4. CSF WCC >30/μl	7/27 (25.9%)	5/138 (3.6%)	25.9% (9.4–42.5%)	96.4% (93.3–99.5%)	7.16 (2.45–20.88)	0.769 (0.61–0.96)
5. Elevated Q_Alb_	18/27 (66.7%)	36/138 (26.1%)	66.7% (48.9–84.4%)	73.9% (66.5–81.2%)	2.56 (1.73–3.76)	0.451 (0.26–0.78)
6. Q_Alb_ >10 × 10^−3^	13/27 (48.1%)	1/138 (0.7%)	48.1%(29.3–67.0%)	99.3% (97.9–100.0%)	**66.44** (9.07–486.92)	0.522 (0.36–0.75)
7. Absence of IgG_IF_ >0% (absence of intrathecal IgG production as defined by Reiber's diagram)	25/27 (92.6%)	66/138 (47.8%)	92.6% (82.7–100.0%)	52.2%(43.8–60.5%)	1.94 (1.58–2.37)	**0.142** (0.04–0.54)
8. Absence of CSF-specific OCB (absence of intrathecal IgG production as defined IEF)	18/27 (66.7%)	8/138 (5.8%)	66.7% (48.9–84.4%)	94.2% (90.3–98.1%)	**11.50** (5.58–23.71)	0.354 (0.21–0.60)
9. Absence of positive MRZ reaction	26/27 (96.3%)	92/138 (66.7%)	96.3% (89.2–100.0%)	33.3% (25.5–41.2%)	1.45 (1.26–1.66)	**0.111** (0.02–0.77)
10. CSF lactate elevated	9/27 (33.3%)	2/138 (1.4%)	33.3% (15.6–51.1%)	98.6% (96.6–100.0%)	**23.00** (5.26–100.6)	0.676 (0.52–0.88)
11. CSF/serum glucose ratio reduced	7/26 (26.9%)	0/138 (0.0%)	26.9% (9.9–44.0%)	100.0%	-	0.731 (0.58–0.92)

95% CI, 95% confidence interval.

IEF, isoelectric focusing; OCB, oligoclonal IgG bands; Q_Alb_, CSF/serum albumin quotient; WCC, white cell count. The bold values indicate most relevant results for PLR (>10) and/or NLR (< 0.02).

**Table 8:** Sensitivity, specificity and likelihood ratios of combinations of basic CSF parameters and/or inflammatory markers to distinguish neurosarcoidosis from MS.

**Parameter**	**Neuro-sarcoidosis, *n*/*N* (%)**	**MS, *n*/*N* (%)**	**Sensitivity, % (95% CI)**	**Specificity, % (95% CI)**	**PLR (95% CI)**	**NLR (95% CI)**	***p*-value**
1. Evidence of 1 of the 3 following parameters: - CSF WCC >30/μl - or Q_Alb_ >10 × 10^−3^ - or absence of CSF-specific OCB	25/27 (92.6%)	14/138 (10.1%)	92.6% (82.7–100.0%)	89.9% (84.8–94.9%)	**9.13** (5.49–15.17)	**0.082** (0.02–0.31)	**<0.001**
2. Combination of a.) 1 of the 3 following parameters: - CSF WCC >30/μl - or Q_Alb_ >10 × 10^−3^ - or absence of CSF-specific OCB and b.) 1 of the 2 following parameters: - Serum sIL-2R elevated - or CSF sIL-2R elevated	10/25 (40.0%)	1/20 (5.0%)	40.0% (20.8–59.2%)	95.0% (85.4–100.0%)	8.00 (1.12–57.35)	0.632 (0.45–0.88)	**0.0066**
3. Combination of a.) 1 of the 3 following parameters: - CSF WCC >30/μl - or Q_Alb_ >10 × 10^−3^ - or absence of CSF-specific OCB and b.) absence of positive MRZ reaction	25/27 (92.6%)	10/138 (7.2%)	92.6% (82.7–100.0%)	92.8% (88.4–97.1%)	**12.79** (6.97–23.43)	**0.080** (0.02–0.30)	**<0.001**

95% CI, 95% confidence interval.

IEF, isoelectric focusing; OCB, oligoclonal IgG bands; Q_Alb_, CSF/serum albumin quotient; WCC, white cell count. The bold values indicate either statistical significant (*p* < 0.05) and most relevant results for PLR (>10) and/or NLR (< 0.02).

Regarding the analysis of single parameters, most inflammatory CSF and serum markers showed high specificity (serum sIL-2R 95.0% [95%-CI 85.4–100.0%], CSF sIL-2R and serum CRP 100.0%), but low sensitivity (serum sIL-2R 40.0% [95%-CI 20.8–59.2%], CSF sIL-2R 38.1% [95%-CI 17.3–58.9%] and serum CRP 25.9% [95%-CI 9.4–42.4%]) and hence high values of respective NLRs (serum sIL-2R 0.63 [0.45–0.88], CSF sIL-2R 0.62 [0.44–0.87] and serum CRP 0.74 [0.83–1.00]) (Table 7). Regarding single basic CSF parameters, highest PLRs were found for Q_Alb_ >10 × 10^−3^ (PLR 66.44 [95%-CI 9.07–486.92]), elevated CSF lactate (PLR 23.00 [95%-CI 5.26-100.6]) and absence of CSF-specific OCB (11.50 [95%-CI 5.58–23.71]) (Table 7). Lowest NLRs were found for absence of positive MRZ reaction (0.11 [95%-CI 0.02–0.77]) and absence of intrathecal IgG synthesis according to Reiber's diagram (0.14 [95%-CI 0.04–0.54]).

In the combined analysis of multiple parameters, the combination of (1) CSF WCC >30/ul, or (2) Q_Alb_ >10 × 10^−3^, or (3) absence of CSF-specific OCB, showed more favorable values for sensitivity (92.6% [95%-CI 82.7–100.0%]), specificity (89.9% [95%-CI 84.8–94.9%]), PLR (9.13 [95%-CI 5.49–15.17]) and NLR (0.082 [95%-CI 0.02–0.31]) to distinguish neurosarcoidosis from MS (Table 8) than the analysis of single parameters (Table 7). When introducing elevated serum or CSF sIL-2R levels as an additional obligatory condition to the latter combination, sensitivity drastically dropped from 92.6% (95%-CI 82.7–100.0%) to 40.0% (95%-CI 20.8–59.2%) and therefore, NLR worsened from 0.082 (95%-CI 0.02–0.31) to 0.63 (95%-CI 0.45–0.88). However, adding absence of positive MRZ reaction (i.e., proof of negative MRZ reaction) as an obligatory condition to the set of basic CSF parameters (i.e., one of the three following parameters fulfilled, (1) CSF WCC >30/ul, or (2) Q_Alb_ >10 × 10^−3^, or (3) absence of CSF-specific OCB) led to an improvement in specificity from 89.9 to 92.8% (95%-CI 88.4–97.1%), in PLR from 9.13 to 12.79 (95%-CI 6.97–23.43) and in NLR from 0.082 to 0.080 (95%-CI 0.02–0.30) while high sensitivity of 92.6% (95%-CI 82.7–100.0) was preserved, marking the best available result regarding diagnostic accuracy in differentiating neurosarcoidosis from MS by CSF analysis.

## 4. Discussion

This retrospective single-center study demonstrates the value of analyzing basic CSF parameters and MRZ reaction to differentiate neurosarcoidosis from MS, which is most commonly considered as a differential diagnosis in routine clinical practice. We show that a CSF WCC >30/μl, Q_Alb_ >10 × 10^−3^ and/or absence of CSF-specific OCB together with absence of positive MRZ reaction are significantly more likely to indicate neurosarcoidosis than MS. As neurosarcoidosis is a serious and often devastating disease that requires therapeutic interventions that differ significantly from those for MS patients, it is of great importance to reliably distuingish between these two diseases and to diagnose the respective disease early and correctly.

Early work on neurosarcoidosis reported that it is the most common disease that can closely mimic MS and is initially misdiagnosed as MS (39). In our cohort of patients with neurosarcoidosis, we also found a high frequency of MS-mimicking neurological syndromes, among them most frequently myelitis, optic neuritis, and sensory and/or motor dysfunction. This is partially in contrast to other reports which find higher frequencies of non-MS mimicking syndromes such as cranial neuropathy in 23–73% (with facial nerve paralysis being the most common) and headache or meningitis in 8–40% (11, 18, 20, 29, 40–47).

According to current literature, 38–69% of patients with neurosarcoidosis do not have a diagnosis of concomitant systemic sarcoidosis before the onset of neurologic symptoms or develop the neurological manifestations as the presenting syndrome of sarcoidosis (17, 20, 46), and 1–20% are reported to have isolated neurosarcoidosis, i.e., without identifiable signs of sarcoid manifestation in other organs (20, 44–51), making the diagnostic situation and differentiation from MS more complicated. The reasons for the large variability in the reported prevalences of neurological symptoms in neurosarcoidosis are probably mainly due to differences in local care and referral between medical specialties and facilities in different countries and centers (11). Another factor could be different fractions of cases with definite, possible, and probable neurosarcoidosis included into the studies and different diagnostic criteria used for inclusion or exclusion of patients. Most recent larger studies report data including 13–25% of reported cases having definite neurosarcoidosis, 59–86% with probable and 15–25% with possible neurosarcoidosis according to Zajicek et al. (18) criteria or Stern et al. (19) criteria (18, 20, 46, 47, 52). Our data included 15% with definite, 70% with probable and 15% with possible neurosarcoidosis and mirroor this typical distribution across recent studies. In addition, recent data suggest that the frequency of neurosarcoidosis among patients with systemic sarcoidosis may probably be higher (up to 34%) than the historically reported 5–15% (11, 47, 52). This finding could be due to heterogeneous awareness of the clinical spectrum of neurosarcoidosis and/or heterogeneous strategies and efforts to prove or disprove the diagnosis of neurosarcoidosis across centers and among different practitioners.

A very interesting and important observation in the routine clinical care of patients with relapsing or re-activated neurosarcoidosis is that neurological deficits often recur in the same way as in previous episodes of disease, due to re-inflammation of the same CNS structures that were previously affected, e.g., re-activation of the same myelitic lesion (11). In one study, disease recurred in 60% of neurosarcoidosis patients who discontinued immunomodulatory therapy with infliximab in the same neuroanatomic lesion (51). This feature of disease recurrence at the same individual neuroanatomical site is less common in MS, as new lesions in individual patients often occur at a wider range of predilection sites. In addition, concomitant involvement of central and peripheral nervous system is very rare in neurosarcoidosis (47), and each of these two manifestations of neurosarcoidosis is associated to different degrees with simultaneouss affections of other organs by sarcoidosis (47). More intensive systematic assessment of cases within neurosarcoidosis-specified registries will most likely enable to assess clearer data (47).

Regarding radiological findings, our cohort of neurosarcoidosis patients showed MRI abnormalities in 89% of cases, most commonly leptomeningeal enhancement (59%). This again reflects typical frequencies reported in literature (69–83% of cases with MRI changes), with the exception, that leptomeningeal enhancement is reported a slightly less frequent (35–46% with leptomeningeal enhancement) (20, 46, 48, 52). Although MRI findings in neurosarcoidosis are highly variable and both contrast-enhancing and non-enhancing lesions are frequently found across various areas of the central nervous system (18, 19), leptomeningeal enhancement is very rare in MS and useful as a rule-in parameter for neurosarcoidosis (53, 54). The frequency of MS-typical lesions found in neurosarcoidosis patients is reported between 5 and 24% (20, 47, 48, 54).

To add another layer of complexity, there is an open debate in the field about coexistence of MS and sarcoidosis in individual patients (54–56). A study analyzing a large epidemiological database found that patients with systemic sarcoidosis have a three times higher risk to additionally acquire the diagnosis of MS during the disease course (57). In one large MS center, retrospective chart review of all neurosarcoidosis patients revealed, that 8/50 cases had been diagnosed with definite or probable MS many years before the diagnosis was changed to neurosarcoidosis because of newly evolving, biopsy-proven systemic sarcoidosis (39). In this study, the misdiagnosis rate of MS instead of true neurosarcoidosis was estimated to be ~1 per 500 (0.2%) patients per decade (39). In contrast, another single-center study described 2/80 patients misdiagnosed with neurosarcoidosis before diagnosis was changed to MS in one case and necrotizing vasculitis in the other case, respectively, as proven by postmortem studies (20). The prevalence of cases with coexistent MS and sarcoidosis stands and falls with the degree of certainty, with which both diseases have been diagnosed. An important starting point would be the identification and documentation of cases with unequivocal histopathological confirmation of both MS and sarcoidosis (i.e., by biopsies of CNS as well as extra-CNS tissue) to better estimate such a prevalence. Until now, such histopathologically double proven cases have not been reported (56), and in all patients with systemic sarcoidosis who underwent brain biopsy for suspected CNS demyelinating disease and are reported in the literature, histopathological analysis revealed neurosarcoidosis (39, 56, 58). The question, whether neurosarcoidosis and MS may co-exist in an individual case, is even less clear, but seems to be much less likely (54, 55).

There have been only few studies assessing typical CSF profiles in neurosarcoidosis patients. Among studies involving more than 20 patients, 32–63% of cases with neurosarcoidosis had a CSF pleocytosis and 46–76% increased CSF protein levels (18, 20, 29, 30). A recent meta-analysis reported increased CSF WCC in 58% and elevated CSF protein in 63% (46). Our findings (64% with pleocytosis and 70.4% with increased Q_Alb_ or CSF protein) are in line with these studies. Conflicting results are reported regarding CSF-specific OCB as a sensitive measure of intrathecal production of IgG in neurosarcoidosis. Recent studies document a very low prevalence of CSF-specific OCB of 3–5% (20, 30), whereas older studies report 27–37% of cases to show CSF-specific OCB (18, 29). This variability is even higher (0–63%) in smaller studies involving < 20 patients (59, 60). In our cohort, 33% of neurosarcoidosis patients had CSF-specific OCB. Since the above-mentioned meta-analysis by Fritz et al. (46) found CSF-specific OCB in 42% of cases with neurosarcoidosis, we think, that a prevalence of 30–40% CSF-specific OCB in neurosarcoidosis is more realistic. Reasons for these differences could be the use of different neurosarcoidosis diagnostic criteria or any other selection bias regarding neurosarcoidosis cohorts over the years in the above-mentioned studies, as well as different IEF methods and reagents for the detection of OCB, resulting in different sensitivities.

Our study shows that these single parameters are not helpful to reliably distinguish neurosarcoidosis from MS, since either PLR was <10 or NLR was >0.1. Values of PLR >10 and NLR < 0.1 are usually regarded as useful (36, 37), as they are increasing the probability for a defined condition by nearly 50% (38). So far, all studies reported these individual parameters without likelihood ratios, and the differences in CSF laboratory findings between neurosarcoidosis and MS remained fuzzy (54). However, we found that combinations of basic CSF parameter, i.e., (1) CSF WCC >30/μl, (2) Q_Alb_ >10 × 10^−3^ or (3) absence of CSF-specific OCB, with or without additional parameters such as increased sIL-2R or negative MRZ reaction, yield PLR values near 10 or >10 and NLR values < 10. These sets of conditional combinations are therefore useful to differentiate between neurosarcoidosis and MS.

The MRZ reaction was first described in 1992 by Felgenhauer and Reiber. It is considered positive, if intrathecally produced IgG react against at least 2 out of 3 viral antigens, i.e., measles (M), rubella (R), and varicella zoster (Z) virus lysates. A positive MRZ reaction is found only in 63% of MS patients, but it is linked with a very high specificity for MS (98%) (61). Reports about the prevalence of positive MRZ reaction in neurosarcoidosis is scarce, and summarizing all cases from literature shows a prevalence of 3/27 (11%) (28, 31–35). Only one study included more than 20 cases with neurosarcoidosis and reported a positive MRZ reaction in 2/22 (9%) of cases (32). We found a lower rate of positive MRZ reaction in both neurosarcoidosis (4%) and MS (33%) than reported in the literature. Again, differences in the diagnostic criteria used and differences in the methodology and use of reagents to detect an MRZ reaction could explain these discrepant results. However, we confirm previous reports, that positive MRZ reaction is significantly higher prevalent in MS than in neurosarcoidosis.

We found a low sensitivity of increased sIL-2R levels in CSF and/or serum for neurosarcoidosis (38–40%). Other studies reported increased levels in more than 50% of patients (62). Although MS patients typically have normal CSF and serum levels of sIL-2R, it is not specific for neurosarcoidosis, as patients with neurotuberculosis, CNS lymphoma and bacterial meningitis (23, 62–64). However, we show that interpreting sIL-2R levels in the context of basic CSF parameters improves PLR and NLR values and therefore diagnostic accuracy in distinguishing neurosarcoidosis from MS. Our data show that additional parameters such as CRP in serum and lactate in CSF are also attractive candidates for inclusion in combined laboratory parameter sets to distinguish neurosarcoidosis from MS.

Since CSF neopterin levels were elevated in 5/5 (100%) of our neurosarcoidosis patients, this may be an additional marker of interest to evaluate in the future for specificity and sensitivity analysis as well as PLR and NLR to distinguish neurosarcoidosis from MS. Unfortunately, neopterin values in CSF were only available in 5 neurosarcoidosis patients and in none of our MS patients, so that a representative analysis of sensitivity and specificity for neurosarcoidosis was not possible. Interestingly, an increase in CSF neopterin has so far been described not only in neurosarcoidosis (65–67) but also in a proportion of MS patients (68). As the literature on neopterin in CSF is sparse in both diseases, this marker should first be systematically analyzed in larger cohorts of neurosarcoidosis and MS patients before being included in further testing for specificity and sensitivity analysis.

We have not analyzed CD4/CD8 T cell ratio in CSF, but an increase of CSF CD4/CD8 T cell ratio >5.0 has so far been found in 14–38% neurosarcoidosis patients and has been proposed as cut-off value to favor the diagnosis of neurosarcoidosis (69–72). Chazal et al. (71) reported that with a lower CSF CD4/CD8 T cell ratio cut-off value of 3.9, the sensitivity was 29% and the specificity 100% for neurosarcoidosis (*n* = 29) vs. MS (*n* = 12), and the sensitivity was 29% and the specificity 87% when comparing neurosarcoidosis with MS and other neuroinflammatory diseases (*n* = 29). Nordström et al. (72) reported that in a cohort of 11 neurosarcoidosis patients and 55 patients with other neurological disease including 6 with MS, an elevated CSF CD4/CD8 T cell ratio ≥5 alone was not adequate for diagnosing neurosarcoidosis (positive predictive value 40%, negative predictive value 88%). However, the combination of a CD4/CD8 T cell ratio ≥5 and elevated lymphocyte count in CSF increased the positive predictive value (57%) with a negative predictive value (88%) and a specificity of 95% for neurosarcoidosis (72). We think, that a more systematic analysis of CSF CD4/CD8 T cell ratio is needed in larger cohorts of neurosarcoidosis and MS patients, to sharpen the picture and to clearly determine the best cut-off value and the respective sensitivity, specificity, PLR and NLR for neurosarcoidosis vs. MS.

There is a dearth of data on PLRs and NLRs of diagnostic tests in general (37). Sensitivities and specificities are insufficient parameters to predict, whether a diagnostic test or a combination of diagnostic tests indicates reliably a certain disease. It is important to note, that PLRs and NLRs are considerably influenced by the prevalence of the respective disease and that they should be reported together with sensitivities and specificities including their respective confidence intervals. By reporting all these measures, diagnostic accuracy is more reliably assessed than by analyzing sensitivities and specificities alone (73). In clinical routine, pretest probabilities or even pretest-odds are often not only unavailable, but also neglected by treating medical team. However, the assessment of likelihood ratios of laboratory parameters and their combinations in relation to clinical and radiological parameters will be invaluable in assessing more accurately the probability that a suspected diagnosis is correct than by intuition or experience. Referencing to known likelihood ratios will most likely help to harmonize test interpretation and diagnostic accuracy between different centers.

It will be important to re-assess these CSF measures in other and larger cohorts. However, from the existing literature including this study a certain pattern of basic CSF parameters seems to emerge, which is typical for neurosarcoidosis, i.e., CSF WCC >30/μl, Q_Alb_ >10 × 10^−3^ and/or absence of CSF-specific OCB together with absence of positive MRZ reaction. If these findings are reproducible in larger cohorts, it should be evaluated whether they can be included in the diagnostic criteria for neurosarcoidosis to distinguish neurosarcoidosis from MS, as this is the most commonly considered differential diagnosis. This type of analysis should also be extended to other neuroinflammatory disease to enable more accurate differential diagnosis.

Furthermore, it would be interesting to combine the above discussed typical patterns of CSF laboratory findings with typical radiological findings such as FDG-PET-positive lymphadenopathy for the analysis of specificity and sensitivity as well as positive and negative likelihood ratios, since FDG-PET-positive lymphadenopathy was found with high prevalence, i.e., in 24/27 (88.9%) neurosarcoidosis patients, and stood out as a sensitive marker among extraneuronal manifestations in our patients with neurosarcoidosis. Unfortunately, we did not have a sufficient number of cases with MS and FDG-PET or CT scan in our cohort to enable us to compare this parameter between neurosarcoidosis and MS patients, as this analysis is not part of the routine assessment in clinical practice. We did not find any studies in the literature that systematically reported the prevalence of lymphadenopathy in untreated MS as identified by means of FDG-PET or CT scan. We found only one observational study assessing signs of lymphadenopathy in MS patients by means of ultrasound (74). This study found that the size and sonographic morphology of deep cervical lymph nodes in 22 drug-free MS patients is significantly different as compared to 20 healthy donors.

Limitations of this study are that the analysis was performed retrospectively and on a single-center level, that the overall number of patients with neurosarcoidosis was low, and that the minority of patients analyzed in this study had a histopathologically proven definite neurosarcoidosis. Analyzing the data retrospectively in principle depends on how accurately clinical, radiological, and laboratory findings are documented in the medical records. Strengths of this study are that we provide the community with robust likelihood ratios for combined sets of CSF parameters, which are useful for routine clinical practice.

## Data availability statement

The raw data supporting the conclusions of this article will be made available by the authors, without undue reservation.

## Ethics statement

Ethical review and approval was not required for the study on human participants in accordance with the local legislation and institutional requirements. The patients/participants provided their written informed consent to participate in this study.

## Author contributions

BV had a major role in the acquisition of the data, analyzed and interpreted the data, and drafted the manuscript for intellectual content. SN acquired, analyzed and interpreted the data, and revised the manuscript for intellectual content. MH analyzed and interpreted the data and revised the manuscript for intellectual content. MZ acquired and analyzed the data, and revised the manuscript for intellectual content. IJ designed and conceptualized the study, acquired, analyzed and interpreted the data, and drafted the manuscript for intellectual content. All authors contributed to the article and approved the submitted version.
