# Supplementary material for: Differentiating neurosarcoidosis from multiple sclerosis using combined analysis of basic CSF parameters and MRZ reaction
Source: Front Neurol. 2023 Mar 24;14:1135392. doi: 10.3389/fneur.2023.1135392 (PMC10080049; doi:10.3389/fneur.2023.1135392)
Supplement: Supplementary file 1 [file Data_Sheet_1.pdf]

Supplementary Material

Differentiating Neurosarcoidosis from Multiple Sclerosis Using Combined Analysis of Basic CSF Parameters and MRZ Reaction

Benjamin Vlad, Stephan Neidhart, Marc Hilty, Mario Ziegler, Ilijas Jelcic*

*** Correspondence:** Corresponding Author: ilijas.jelcic@usz.ch

Supplementary Table 1. Overview of biopsied tissue of neurosarcoidosis patients

	n/N (%)
**Tissue biopsied in cases with definite neurosarcoidosis**	
- Meninges	1/4 (25.0%)
- Cerebellum	1/4 (25.0%)
- Combined meningeal and brain biopsy	1/4 (25.0%)
- Optic nerve	1/4 (25.0%)
**Tissue biopsied in cases with probable neurosarcoidosis**	
- Pulmonary hilar lymph nodes	16/19 (84.2%)
- Extrapulmonary lymph nodes*	3/19 (15.8%)

*cervical lymph nodes in 2/19 (10.5%), inguinal lymph node in 1/19 (5.3%)

Supplementary Table 2. Review of frequencies of elevated basic CSF parameters in patients with neurosarcoidosis among studies reported in the current literature. The frequencies of patients with definite versus probable/possible neurosarcoidosis, elevated CSF white cell count (WCC), elevated CSF protein or elevated Q_{Alb}, and CSF-specific oligoclonal bands (OCB) indicating intrathecal immunoglobulin synthesis are shown.

**Study (reference)**	**Definite vs. probable/possible neurosarcoidosis, n (%) / n (%)**	**CSF WCC elevated, n/N (%)**	**CSF protein or Q_{Alb} elevated, n/N (%)**	**CSF-specific OCB, n/N (%)**
Borucki et al., 1989	0 (0%) / 5 (100%)	4/5 (80.0%)	n.r.	0/5 (0%)
McLean et al., 1990	19 (100%) / 0 (0%)	n.r.	n.r.	12/19 (63.2%)
Zajicek et al., 1999	8 (12.9%) / 54 (87.1%)	34/62 (54.8%)	45/62 (72.6%)	20/54 (37.0%)
Joseph and Scolding, 2009	5 (22.7%) / 17 (77.3%)	7/22 (31.8%)	10/22 (45.5%)	3/11 (27.3%)
Kidd, 2018	n.r.	45/89 (50.6%)	68/89 (76.4%)	4/89 (4.5%)
Arun et al., 2020	11 (12.8%) / 75 (87.2%)	47/75 (62.7%)	45/67 (67.2%)	2/70 (2.9%)
Current study	4 (14.8%) / 23 (85.2%)	15/27 (55.6%)	19/27 (70.4%)	9/27 (33.3%)

n.r. – not reported

Supplementary Table 3. Mean values of parameters indicating intrathecal production of IgG, IgA or IgM according to Reiber's diagram.

Parameter	Overall	Neuro-sarcoidosis	MS	p-value
**a) Mean CSF/serum quotient of IgG, IgA or IgM**				
mean Q_{IgG} (SD)	5.3 (5.0)	9.3 (10.2)	4.5 (2.5)	**0.024**
mean Q_{IgA} (SD)	3.0 (6.1)	8.9 (13.5)	1.8 (1.9)	**0.014**
mean Q_{IgM} (SD)	1.3 (4.2)	4.1 (9.9)	0.7 (1.1)	0.093
**b) Mean intrathecal fraction of IgG, IgA or IgM in patients with intrathecal production of IgG, IgA or IgM (IgG/A/M_{IF} > 0%)**				
mean IgG_{IF} (SD), if IgG_{IF} > 0%	38.9 (18.4)	39.3 (3.2)	38.9 (18.6)	0.897
mean IgA_{IF} (SD), if IgA_{IF} > 0%	42.4 (25.2)	32.1 (26.3)	47.5 (25.2)	0.246
mean IgM_{IF} (SD), if IgM_{IF} > 0%	46.4 (21.0)	48.3 (16.2)	46.1 (22.5)	0.836

SD – standard deviation

Supplementary Table 4. Frequency of patients with intrathecal antiviral IgG production. Intrathecal production of IgG against a specific viral antigen is present, if CSF/serum antibody index (CAI) is ≥ 1.5. Frequencies are shown for the single virus-specific antibody reactivity species, measles (M), rubella (R) and varicella zoster (Z) virus antigens, and the combinations of these (M+R, R+Z, M+Z and M+R+Z).

**Parameter**	**Overall**	**Neuro-sarcoidosis**	**MS**	**p-value**
0/3 antiviral CAI values > 1.5, n (%)	75/165 (45.5%)	25/27 (92.6%)	50/138 (36.2%)	**<0.001**
1/3 antiviral CAI values > 1.5 (M or R or Z), n (%)	43/165 (26.1%)	1/27 (3.7%)	42/138 (30.4%)	**0.008**
2/3 antiviral CAI values > 1.5 (M+R or R+Z or M+Z), n (%)	25/165 (15.2%)	0/27 (0.0%)	25/138 (18.1%)	**0.035**
3/3 antiviral CAI values > 1.5 (M+R+Z), n (%)	22/165 (13.3%)	1/27 (3.7%)	21/138 (15.2%)	0.194

Supplementary Table 5. Mean values of measles-, rubella- and zoster-specific CSF/serum antibody index (CAI) values. Intrathecal production of IgG against a specific viral antigen is present, if CSF/serum antibody index (CAI) is ≥ 1.5

**Parameter**	**Overall**	**Neuro-sarcoidosis**	**MS**	**p-value**
Measles (M)-specific CAI, mean (SD)	2.1 (6.1)	1.1 (2.3)	2.3 (6.6)	0.092
Rubella (R)-specific CAI, mean (SD)	2.2 (3.4)	1.1 (1.2)	2.4 (3.7)	**0.001**
Zoster (Z)-specific CAI, mean (SD)	2.9 (5.4)	1.4 (2.8)	3.2 (5.7)	**0.018**

SD – standard deviation

Supplementary Table 6. Review of frequencies of positive MRZ reaction among patients with neurosarcoidosis reported in the current literature.

**Study (reference)**	**Frequency of positive MRZ reaction[1], n/N (%)**
Felgenhauer and Reiber, 1992	0/1 (0%)
Reiber et al., 1998	<1%[2]
Graef et al., 1994	0/1 (0%)
Hottenrott et al., 2015	2/22 (9.1%)
Brecht et al., 2012	0/2 (0%)
Endres et al., 2022	1/1 (100%)
Overall	3/27 (11.1%)

[1]positive MRZ reaction defined as intrathecal production of IgGs reactive against at least two of three antigens, measles (M), rubella (R) and varicella zoster (Z) virus antigens, i.e, M+R or M+Z or R+Z or M+R+Z.
[2]the frequency of triple-positive MRZ reaction (intrathecal synthesis of IgG specific for M+R+Z) was 0.01%; but since the absolute patient numbers are not mentioned by the authors, they are not included in the overall statistical analysis.
